# Distinctive Gene Expression Profiles and Effectors Consistent With Host Specificity in Two Formae Speciales of *Marssonina brunnea*

**DOI:** 10.3389/fmicb.2020.00276

**Published:** 2020-03-10

**Authors:** Fei Ren, Dong-Hui Yan, Guanghua Wu, Xiaoming Sun, Xiaoyu Song, Ruhua Li

**Affiliations:** ^1^Research Institute of Forest Ecology, Environment and Protection, Key Laboratory of Forest Protection Affiliated to State Forestry and Grassland Administration of China, Chinese Academy of Forestry, Beijing, China; ^2^Institute of Cereal & Oil Science and Technology, Academy of National Food and Strategic Reserves Administration, Beijing, China

**Keywords:** *Marssonina brunnea*, forma specialis, poplar, disease, host specificity, *de novo* transcriptome, effectors

## Abstract

The knowledge on the host specificity of a pathogen underlying an interaction is becoming an urgent necessity for global warming. In this study, the gene expression profiles and the roles of effectors in host specificity were integrally characterized with two formae speciales, multigermtubi and monogermtubi, of a hemibiotrophic pathogen *Marssonina brunnea* when they were infecting respective susceptible poplar hosts. With a functional genome comparison referring to a *de novo* transcriptome of *M. brunnea* and Pathogen–Host Interaction database functional annotations, the multigermtubi strain showed abundant and significant differentially expressed unigenes (DEGs) (more than 40%) in colonizing the initial invasion stage and in the necrotrophic stage. The monogermtubi strain induced less than 10% of DEGs in the initial invasion stage but which abruptly increased to more than 80% DEGs in the necrotrophic stage. Both strains induced the least DEGs in the biotrophic stage compared to the initial invasion and necrotrophic stages. The orthologs of the effector genes *Ecp6*, *PemG1*, *XEG1*, *ACE1*, and *Mg3LysM* were exclusively induced by one of the two formae speciales depending on the infection stages. Some unigenes homologous to carbohydrate lytic enzyme genes, especially pectate lyases, were notably induced with multigermtubi forma specialis infection but not expressed in the monogermtubi forma specialis at an earlier infection stage. The extraordinary differences in the functional genome level between the two formae speciales of *M. brunnea* could be fundamental to exploring their host specificity determinant and evolution. This study also firstly provided the fungal transcriptome of the monogermtubi forma specialis for *M. brunnea*.

## Introduction

The host specificity of a pathogen is becoming a much urgent question to be uncovered in that global climate change will make pathogens jump or broaden their host specificity to become emerging diseases (Poloni and Schirawski, 2016; Inoue et al., 2017; Borah et al., 2018). Gathered evidences suggest that gene expression profiles and virulent factors like effectors could be associated with pathogenic host specificity (Parkinson et al., 2016; Poloni and Schirawski, 2016; Inoue et al., 2017; Palma-Guerrero et al., 2017). However, the determinant mechanism underlying host specificity is still waiting to be elucidated (Borah et al., 2018; Frantzeskakis et al., 2019; Morris and Moury, 2019). Yet most of such knowledge at present has been obtained through documents on strains or formae speciales of pathogens against phylogenetically distant hosts (Borah et al., 2018; Frantzeskakis et al., 2019; Morris and Moury, 2019).

The fungal species *Marssonina brunnea* (Ell. et Ev.) Magn. [sexual morph: *Drepanopeziza brunnea* (Ellis & Everh.) Rossman & W.C. Allen (Rossman et al., 2018)] is the most pathogenic species causing the marssonina foliar anthracnose diseases on poplars (Spiers, 1984; Erickson et al., 2004; Zhu et al., 2012; Chen et al., 2015; Call and St. Clair, 2017). It often severely breaks out in poplar plantations all over the world recently and threatens poplar crops for the future (Erickson et al., 2004; Zhu et al., 2012; Call and St. Clair, 2017; Zhang et al., 2018a). The population of the pathogen has been verified to be differentiated into two host-adapted formae speciales in the field based on host ranges, pathogenicity, and phylogenetic nature (Spiers, 1984; Han et al., 1998a, 2000; Sun et al., 2015; Call and St. Clair, 2017; Zhang et al., 2018b). In China (Han et al., 1998a, b, 2000; Sun et al., 2015; Zhang et al., 2018b), one forma specialis, *M. brunnea* f. sp. *monogermtubi* (MONO), induces a disease on susceptible poplar hosts strictly limited in section Leuce in the *Populus* genus. The other forma specialis, *M. brunnea* f. sp. *multigermtubi* (MULT), infects susceptible hosts strictly limited in sections Aigeiros and Tacamahaca. Cross-infectivity on hosts among the sections mentioned above does not happen on each forma specialis in the field (Spiers, 1989; Han et al., 2000). However, previous reports showed that the two formae speciales shared a very highly similar infection process in respective susceptible hosts living a hemibiotrophic lifestyle (Zhang et al., 2018a, b). Therefore, the two formae speciales can be an ideal model for gaining an insight into the mechanisms of host specificity. The MULT fungi were, to some extent, recorded with gene expressions due to their poplar hosts being the worldwide most commercially important poplar crops until nowadays (Yu et al., 1997; Zhang et al., 2007, 2018b; Yuan et al., 2008; Cheng et al., 2010; Zhu et al., 2012; Jiang et al., 2014; Chen et al., 2015; Xing et al., 2018; Jiang et al., 2019). For example, the MULT strains induced many LysM effectors during infection (Yuan et al., 2008; Cheng et al., 2010; Zhu et al., 2012; Jiang et al., 2014; Chen et al., 2015; Zhang et al., 2018b). However, it is unclear whether its gene expression regulation and virulent factor production are associated with host specificity to the MULT forma specialis. In contrast, no correlating data on transcriptome and/or secretome data including effectors have been characterized for the forma specialis MONO, another forma specialis of *M. brunnea*, although the pathogenesis of the MULT fungi was evolved from the MONO fungi (Han et al., 1998a, b; Sun et al., 2015).

We assumed that the host-specific diversification between the two formae speciales was determined with their infection of different molecular events. Here, we report the underlying molecular mechanisms of host specificity through a comparative functional genome approach. This is the first report on host-specific differentiation between formae speciales that infect phylogenetically close hosts from various subgenera in the same genus and have a closely similar infection process. In addition, it is also the first time to provide knowledge on the gene expressions in the transcriptomic characterization of molecular interactions between the monogermtubi pathogens and their poplar hosts.

## Materials and Methods

### Fungal Strains and Infecting Process

In this study, the strain MONO isolated from *Populus tomentosa* Carr. was used to represent as forma specialis of *M. brunnea* f. sp. *Monogermtubi*, which infects poplar hosts in section Leuce of the *Populus* genus. The strain MULT isolated from *Populus nigra* L. was used as a representative of the forma specialis of *M. brunnea* f. sp. *multigermtubi* infecting poplars in section Aigeiros. The host specificity of the two strains had been investigated among various hosts from different sections in the *Populus* genus (Zhang et al., 2018a, b).

According to pilot tests, one hybridized variety *Populus alba* × *P. alba* var. *pyramidalis* in section Leuce was sensitive to MONO forma specialis. A variety of *Populus deltoids* cv. “Zj-2” from section Aigeiros served as a sensitive host of MULT forma specialis (Zhang et al., 2018a, b). The healthy and mature leaves from the host seedlings of the cultivar varieties were used for pathogen inoculation. The leaves were inoculated with conidia suspension at a concentration of 1 × 10^6^ conidia/ml and sampled at 6, 36, and 96 h post-inoculation (hpi) with consideration of biological triplicates (Zhang et al., 2018a, b). The three sampling time points presented an initial invasion stage with appressoria evidences at 6 hpi, an asymptomatic biotrophic life phase with haustoria-like structures at 36 hpi, and a necrotrophic life stage with initial lesion spots at 96 hpi, respectively, covering a whole hemibiotrophic infection period for the pathogens (Zhang et al., 2018a, b). After inoculation, the rest of the conidia suspensions were synchronously cultured in sterile water at 25°C under the same conditions as with the inoculated leaves. The germinating samples of the conidia incubated in sterile water for 6 h with triplicates were used as an initial transcript control for those pathogens during the infection course. All of the samples were frozen by liquid N immediately after sampling and stored at −80°C until RNA extraction.

### RNA Extraction, Library Preparation, and Sequencing

Total RNA from all infected leaves at each sampling time point and the control samples with biological triplicates were extracted separately using TRIzol (Invitrogen) following the manufacturer’s procedure (Zhang et al., 2018b). One fraction of total RNA for each sample was used for RNA-seq analysis, and the rest of the samples were used for transcriptome data verification by real-time PCR. The RNA with the highest quality of biological replicate samples for each time point and the control conidia at 6 h were used for library preparation and sequencing. Library preparations were performed *via* the manufacturer’s procedures with NEBNext Ultra TM RNA Library Prep Kit (Illumina Inc.) by Biomarker Company (Beijing, China). The first-strand cDNA was synthesized with a random hexamer primer and M-MuLV reverse transcriptase (NEB, United States). The second-strand cDNA was synthesized with DNA Polymerase I and RNase H. The size adaptor-ligated cDNA was selected with 3 μl USER Enzyme (NEB, United States) at 37°C for 15 min, followed by 5 min at 95°C. PCR was performed on the cDNA with Phusion High-Fidelity DNA polymerase, Universal PCR primers, and Index (X) Primer. The cDNA library preparations were sequenced on an Illumina sequencing platform (Illumina Hiseq 2500, Illumina, San Diego, CA, United States).

### *De novo* Transcriptome Assembly and Analysis

Raw Illumina reads were processed to remove reads containing adapter, poly-N (>5% N), and low quality (>50% Ophred score 20, <80% Q30) through in-house perl scripts (Biomarker Company, Beijing). The clean reads retrieved from the raw reads above were used for all the other downstream analyses. As the infected leaf samples contained both poplar and *M. brunnea* transcriptomes, the clean reads were aligned with the *Populus trichocarpa* v 3.0. Genome (Phytozome), unmapped clean reads for the pathogen were retrieved by using TopHat version 2.0.6. Furthermore, the unmapped clean reads from MONO were found to be less than 10% mapping to the reference genome of strain M6 from *M. brunnea* f. sp. *multigermtubi* for *M. brunnea* fungi (Zhu et al., 2012). Therefore, a *de novo* transcriptome approach was assembled for this study purpose. All clean reads from both formae speciales, which were unaligned to the *P. trichocarpa* genome as well as reads from the conidia control, were treated as a pool of reads. This pool of reads was *de novo* assembled into a reference transcriptome for further analysis using the Trinity software (release-2013-11-10) with a default k-mer length of 25. The reliability of the experimental replicates was assessed using SERE (Schulze et al., 2012).

BLASTX programs performed unigene searches against the sequences in the NCBI non-redundant (Nr) protein database, Swiss-Prot, Kyoto Encyclopedia of Genes and Genomes (KEGG), Clusters of Orthologous Group (COG), and Gene Ontology (GO) (the *e*-value cutoff was 1.0E-5). Unigenes were tentatively identified according to top hits against known sequences. The resulting unigenes were used as references for the determination of GO and COG functional classification and were analyzed further using the KEGG database. Gene function was annotated based on the following databases: Nr, Nt (NCBI non-redundant nucleotide sequences), Pfam (Protein family), KOG/COG, Swiss-Prot, KO, and GO.

### Different Expression Analysis of Unigenes

The sequence reads from each infected sample and the conidia control sample were mapped to the unigenes originating *de novo* transcriptome assembled from all the MONO and MULT samples with Tophat 2.06 under standard parameters. The mapped number of reads was calculated using HTseq-count. Unigene expression levels were calculated as FPKM values using the program Cufflinks. DESeq R package was used to identify differentially expressed genes (DEGs) with a *p*-value ≦ 0.05 and a | log2 (fold change) | ≧ 2 as the threshold. GO enrichment implemented analysis on biological function for the DEGs by the GOseq R packages-based Wallenius non-central hyper-geometric distribution. The statistical enrichment of the DEGs in the KEGG pathway was implemented for metabolic pathways by KOBAS software.

The raw data to generate all clean data used for this study were deposited in NCBI with Bioprojects accession number PRJNA395840.

### Verification of the RNA-seq by qRT-PCR

Nine genes selected from significantly different expression unigenes were used for the qRT-PCR verification of the transcriptome. The unigenes and their specific primers are listed in Supplementary Table S1. The total RNA samples from the corresponding control and infected RNA samples collected at different times for RNA-seq were reversely transcribed into cDNA in a 20-μl reaction mixture volume using the PrimeScript RT Master Mix Kit (TaKaRa, China) following the recommended procedure. The qRT-PCR was executed on a LightCycler (Roche, German) with the kit of SYBR Premix Ex Taq II (TaKaRa, China) according to the manufacturer’s protocol. The qRT-PCR reaction processes were under the following conditions: initial denaturation at 95 for 30 s, followed by 40 cycles of 95 for 5 s, and 60 for 30 s. All amplification reactions were run in triplicate with three biological replicates. The expression levels of the target genes of the pathogens in the infection process were normalized to the constitutively expressed actin gene (reference gene) and were calibrated compared with the levels recorded in the control samples (set as 1) using the 2^–ΔΔCt^ method (Livak and Schmittgen, 2001).

### Candidate Effector and Secretory Genes Analysis

OrfPredictor software predicted the ESTs of the transcripts with differential up-regulated genes during infection and translated the ESTs into amino acid sequences (Min et al., 2005). The predicted proteins as queries hit secreted proteins in Fungal Secretomes of FunSecKB2 database with Blastp at value ≦ 1E-5 (Meinken et al., 2014). The same predicted proteins were also analyzed in the EffectorP 2.0 beta database for predicting the candidate effector genes (Dodds et al., 2016). Those up-regulated genes shared in both databases were taken as putative effector genes; otherwise, they were taken only as secretory proteins or candidate effectors. The roles of the secretory proteins or effectors were annotated in the Pathogen–Host Interactions database (PHI-base^1^).

## Results

### *De novo* Transcriptome Assembly and Functional Annotation for *M. brunnea* Transcriptome

**FIGURE 1:**
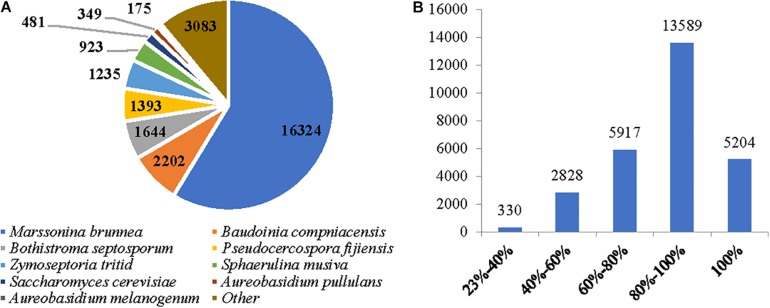
The assembled sequence quality of the *de novo* transcriptome for two *M. brunnea* formae speciales. **(A)** The unigene number of the contributing microorganism species. **(B)** An identity distribution for unigenes.

RNA sequencing for the native *M. brunnea* on the Illumina HiSeq 2500 platform generated a total of 779,966,044 clean reads (Supplementary Table S2). The *de novo* assembled transcriptome resulted in 44,011 unigenes with more than 200 bp length sequences (Supplementary Figure S1). The MULT and MONO unigenes were mapped more than 70% in quality with the *de novo* assembled transcriptome (Supplementary Table S3). *M. brunnea* was the most frequent organism in the Blast hits with 16,324 unigenes (58.70%), followed by *Baudoinia compniacensis* with 2,202 unigenes (7.92%) and *Dothistroma septosporum* with 1,644 unigenes (5.91%), etc. (Figure 1A). An identity distribution analysis (Figure 1B) revealed that 5,204 (18.67%) hits showed 100% similarity to their query sequences, 13,589 (48.76%) hits had a similarity range from 80 to 99% identities, 5,917 (21.23%) hits had similarity ranging between 60 and 79% identities, 2,828 (10.15%) hits had a similarity ranging from 40 to 59% identities, and 330 (1.18%) hits had a similarity range from 23 to 40%. The total number of annotated unigenes ranged from 9,606 (34.31%) to 27,869 (99.56%) in this *de novo* transcriptome against those in various annotation databases including COG, GO, KEGG, KOG, Swiss-Port, eggnog, and NCBI-NR (Supplementary Table S4).

**FIGURE 2:**
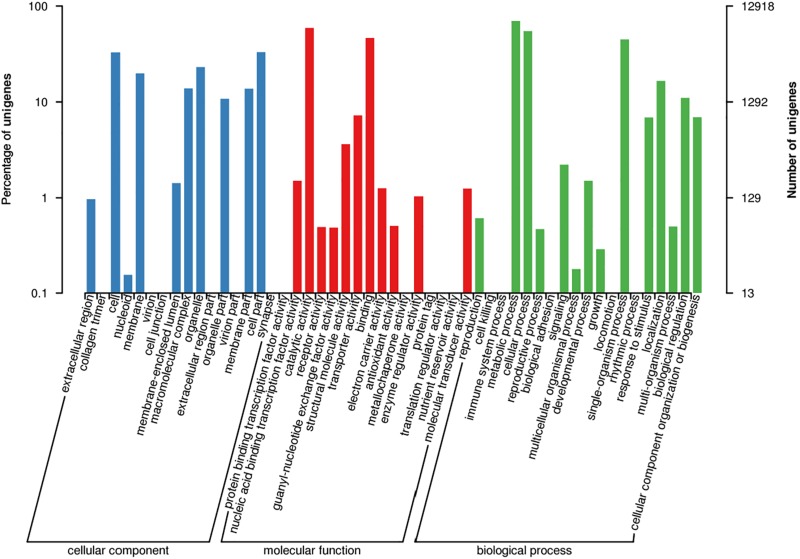
Functional annotation for unigenes in the *de novo* transcriptome of *M. brunnea* with gene ontology (GO) terms.

**FIGURE 3:**
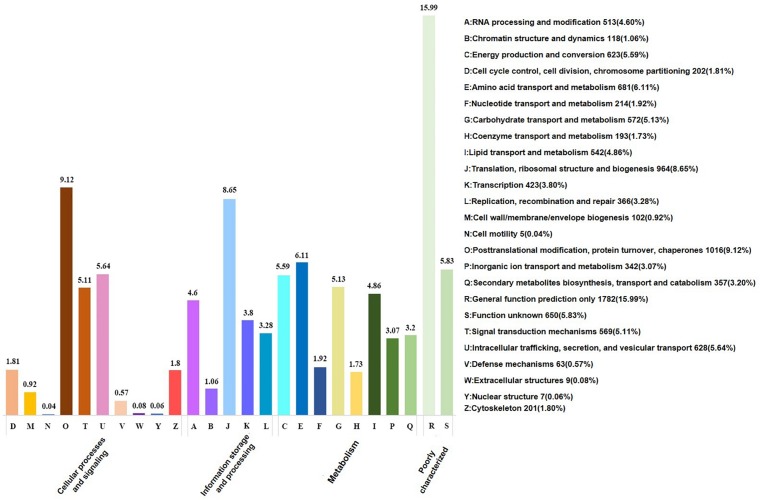
KOG functional annotation on the *de novo* transcriptome of *M. brunnea*.

According to GO term enrichment analyses, the 12,918 unigenes were classified and occurred in three Blast2GO categories of biological process, cellular component, and molecular function (Supplementary Table S4). Among the 12,918 unigenes, 11,670 unigenes were clustered with the “biological process” terms, in which metabolic processes were found to have the most abundant unigenes, followed by “molecular function” and “single-organism process” terms (Figure 2 and Supplementary Table S5). The “molecular function” term hosted 8,535 unigenes, which were highly represented with catalytic activity and binding categories. The “cellular component” term had 5,912 unigenes with the most abundance in cell, cell part, and organelle (Figure 2). A search against the KOG database resulted in the assignment of KOG classification for 12,512 unigenes (Supplementary Tables S4, S5). Among them, 11,142 unigenes were grouped into four KOG functional categories and 25 specific function classes (Figure 3 and Supplementary Table S6). The KOG functions of 11,142 unigenes were distributed as 2,802 (25.15%), 2,384 (21.40%), 3,542 (31.79%), and 2,432 (21.83%) unigenes into “cellular processes and signaling,” “information storage and processing,” “metabolism,” and “poorly characterized” categories, respectively (Figure 3). The top five KOG function classes with the most unigene quantity were “R: general function prediction only” (1,782, 15.99%), “O: posttranslational modification, protein turnover, chaperones” (1,016, 9.12%), “J: translation, ribosomal structure, and biogenesis” (964, 8.65%), “E: amino acid transport and metabolism” (681, 6.11%), and “S: function unknown” (650, 5.83%) (Figure 3). Therefore, besides the poorly characterized category of the unigenes in the cluster of the general function prediction only (R) and function unknown (S), the *de novo* transcriptome was characterized with the numerical transcripted unigenes in posttranslational modification (O) for cellular processes and signaling, in translation, ribosomal structure, and biogenesis (J) for cellular processes and signaling, and in amino acid metabolism (E) for metabolism (Figure 3). Furthermore, relatively abundant unigenes were also found in carbohydrate (G) and lipid (I) transport and metabolism KOG classes (Figure 3). The classes for “N: cell motility,” “W: extracellular structures,” and “V: defense mechanisms” were the smallest groups observed in the KOG annotation analysis with 9, 9, and 63 unigenes, respectively.

### The Differentially Expressed Genes Profiles With the Two *M. brunnea* Formae Speciales Infecting Hosts

**FIGURE 4:**
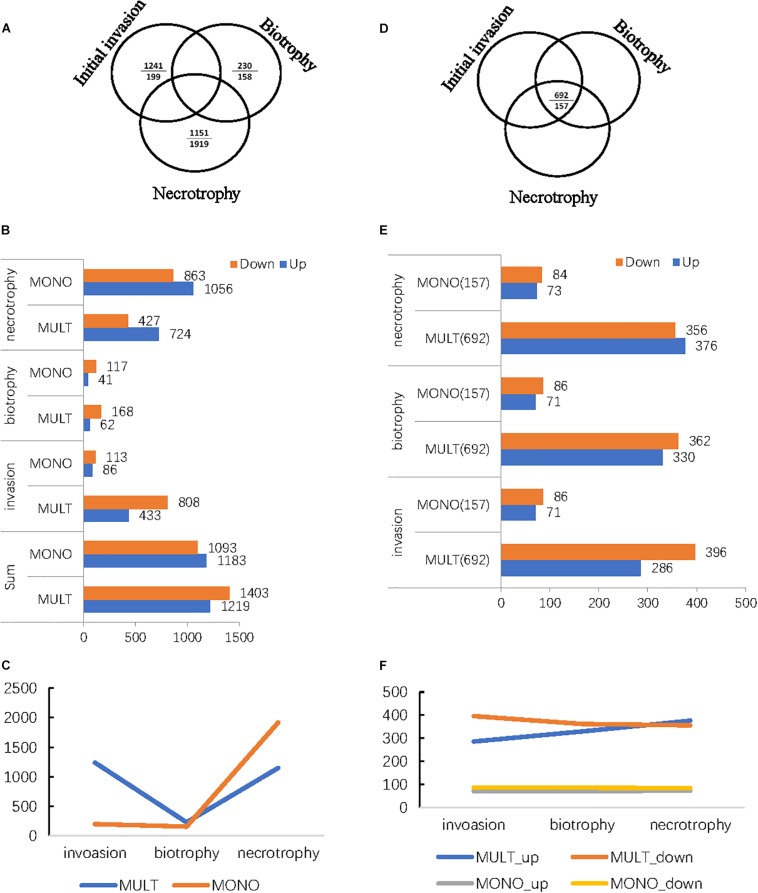
The differentially expressed genes (DEGs) profiles of the two *M. brunnea* formae speciales MULT and MONO infecting the respective susceptible hosts in three infection stages and in common throughout the whole infection course. **(A)** The number of DEGs expressed specific to the infection time (stage). Relative to the short dash, the upper entry is the number of MULT and the entry below is the number of MONO. **(B)** The number of DEGs with a change in direction, up or down, in distribution relative to each infection time (stage). **(C)** DEGs profiles specifically expressed to *M. brunnea* formae speciales. **(D)** The common expressed number of DEGs throughout the infection course. **(E)** The common expressed number of DEGs distribution to each infection time. **(F)** The common expressed number of the DEGs change trend to *M. brunnea* formae speciales.

MULT and MONO strains induced 3,314 and 2,433 significantly differentially expressed unigenes (DEGs) (Figure 4), respectively. Among the DEGs, the two strains had a close number of DEGs specifically expressed to three infection times (6, 36, and 96 hpi), with 2,622 DEGs in MULT and 2,276 DEGs in MONO (Figures 4A,B), but the number of DEGs expressed among the three infecting times were changed in a significantly different pattern between the two strains (Figure 4B). Furthermore, for each infection stage (time)-specific DEG, the strain MULT expressed 47.3% (1,241) DEGs in the initial invasion stage (at 6 hpi), 8.8% (230) DEGs in the biotrophic stage (at 36 hpi), and 43.9% (1,151) DEGs in the necrotrophic phase (at 96 hpi); the strain MONO expressed 8.8% (199), 6.9% (158), and 84.3% (1,919) DEGs for each corresponding stage as mentioned above, respectively (Figure 4B). Therefore, based on the transcript expressed profiles, MULT was found to dramatically decrease in terms of the number of DEGs at the biotrophic stage compared to that at the initial invasion or the necrotrophic phases, showing a V-shape in the DEGs expression profile (Figure 4C). Yet MONO had a remarkable feature of abruptly increasing the number of DEGs at the necrotrophic stage, showing an oblique mirror L-shape to express the gene profiles (Figure 4C). In addition, both strains had a comparatively obvious difference in the number of DEGs expressed commonly through their own whole infection course. MULT and MONO had 692 and 157 common DEGs (Figure 4D), respectively. Furthermore, following the three infection times, MULT increased by 90 common up-regulated DEGs and decreased by 40 common down-regulated unigenes in the necrotrophic stage compared to that in the initial invasion stage, while MONO almost held a few changes in common DEGs number shift with only two unigenes changing expression direction (Figures 4E,F).

The RNA-seq datasets of three biological replicates of the samples from each sampling time point and the conidial control samples were able to be closely grouped in clusters with good correlation (Supplementary Figure S3). Furthermore, the transcriptome was validated with nine unigenes randomly selected from MULT and MONO. Each of the nine unigenes was behaviorally expressed in a consistent trend between qRT-PCR and Illumina RNA-seq platforms (Supplementary Table S1 and Supplementary Figure S2).

### The Dominant Functions of the Up-Regulation Genes Related to Forma Specialis

MULT had a total of 1,219 up-regulated DEGs specific to three infection times (6, 36, and 96 hpi) and 277 up-regulated DEGs common during the infection course. MONO had 1,183 up-regulated DEGs specific to the three times and 70 up-regulated DGEs common to the three times (Figures 4B,E and Table 1). The up-regulated unigene distribution of each strain is shown in a numerical statement with functional annotation in Table 1. Each forma specialis induced a unique set of significantly expressed unigenes, which had few unigenes shared with the other forma specialis, in each infection stage (Table 1). Moreover, in the initial invasion stage, MULT expressed 428 (83.3%) up-regulated unigenes; MONO induced 81 (15.8%) unique ones. Only five (1%) up-regulated unigenes were shared by them. In the biotrophic stage, 61 (59.8%) to MULT, 40 (39.2%) to MONO, and only 1 (1%) unigene was shared by both; in the last stage of necrotrophy, 690 (39.5%) to MULT, 1,022 (58.5%) to MONO, and 34 (1.9%) unigenes were shared by the two formae speciales (Table 1). The fact that less than 2% of the up-regulated DEGs were shared by the two strains at each infection time distinguished the transcriptomes between the MULT and the MONO strains (Table 1). For the common up-regulated unigene through the whole infection course, MULT induced 229 (76.6%) up-regulated DEGs associated with host specificity, MONO had 22 (7.4%) up-regulated DEGs specifically expressed in its host, and 48 (16.1%) expressed unigenes were shared by both formae speciales (Table 1).

**TABLE 1:** The numerical statement of the up-regulated unigenes in infection stages and functional classes with eggNOG and NR annotation for the two formae speciales of *M. brunnea*.

Annotation	Databases		Invasion			Biotrophy			Necrotrophy			Common		
			MU	MO	MU + MO	MU	MO	MU + +MO	MU	MO	MU + MO	MU	MO	MU + MO
Annotated	eggNOG	A	5	2	0	1	0	0	15	18	1	1	0	1
		B	2	0	0	0	0	0	3	0	0	0	0	0
		C	18	3	0	3	0	0	10	17	0	0	0	1
		D	3	0	0	1	0	0	18	6	1	0	0	0
		E	12	2	0	1	0	0	19	34	1	1	0	0
		F	2	0	0	1	0	0	7	5	0	0	0	0
		G	20	1	0	1	0	0	31	46	0	10	0	2
		H	2	0	0	1	0	0	7	8	0	1	0	0
		I	20	0	0	0	0	0	9	15	1	0	0	0
		J	17	0	0	7	0	0	22	29	1	2	0	1
		K	9	1	0	4	3	0	12	18	1	2	1	2
		L	5	1	0	0	1	0	19	8	1	0	0	0
		M	5	0	0	0	0	0	1	5	0	1	1	0
		N	0	0	0	0	0	0	0	1	0	0	0	0
		O	26	3	1	2	3	0	17	21	5	3	0	0
		P	10	0	1	1	0	0	7	12	0	1	0	1
		Q	15	2	0	6	0	0	6	39	1	3	0	0
		R	64	14	2	9	4	0	90	166	6	42	2	3
		S	92	14	0	10	10	0	142	184	10	15	1	2
		T	15	3	0	0	0	0	12	24	0	0	0	0
		U	10	3	0	0	0	0	3	3	1	2	0	0
		V	1	0	0	0	0	0	1	6	0	0	0	0
		W	1	0	0	0	0	0	2	1	0	0	0	0
		Z	7	0	0	0	1	0	10	5	1	1	0	1
	NR		43	24	1	9	8	0	127	180	1	42	1	3
	Sum		404	73	5	57	30	0	590	851	32	127	6	17
Unannotated			24	8	0	4	10	1	100	171	2	102	16	31
Total number			428	81	5	61	40	1	690	1,022	34	229	22	48

The eggNOGs and NR databases were applied for analyzing the functions of the up-regulated unigenes to annotate the maximum number of unigenes for both fungi (Table 1 and Supplementary Table S4). According to the eggNOG function annotation (Table 1), the two KOG functional categories, the general function prediction only (R), and the function unknown (S) were clustered with the most up-regulated strain-specific DEGs induced by both formae speciales at each infection time or in common through the infection courses. Posttranslational modification (O) was the following functional class with MULT (6.1%, 26/428) and from MONO (3.7%, 3/81). Other dominant functional classes for strain-specific up-regulation unigenes depended on the infection stages (Table 1). In the initial invasion stage, MULT induced up-regulated unigenes that functioned dominantly in carbohydrate transport and metabolism (G) (4.6%, 20/428), lipid transport and metabolism (I) (4.6%, 20/428), and energy production and conversion (C) (18/428); MONO induced unigenes mainly in energy production and conversion (C) (3/81), signal transduction mechanisms (T) (3/81), and intracellular trafficking and secretion (U) with 3.7% (3/81) unigenes. Actually, in this invasion infection stage, MULT induced much more up-regulated DEGs than MONO in most KOGs functional classes, especially in the metabolism categories for amino acid (E), carbohydrate (G), secondary metabolites (Q), and energy (C), as well as in cellular processes and signaling for posttranslational modification, protein turnover, and chaperones (O) (Table 1). Furthermore, MULT strain employed significantly more up-regulated DEGs functioning as degrading enzymes on carbohydrate, protein, and lipid metabolisms than the MONO strain in the initial colonization, which contributed to the pathogenicity to infect hosts, such as pectate lyase, cutinase, subtilase, etc. (Supplementary Table S7-invasion). In contrast, the up-regulated DEGs of MONO were found to have only two protease unigenes annotated with KOG and NR databases (Supplementary Table S7-invasion). Even MONO had not any up-regulated DEGs annotated in some functional classes such as lipid transport and metabolism (I) and translation, ribosomal structure, and biogenesis (J) in which MULT expressed 17 and 20 DEGs, respectively (Table 1).

In the biotrophic stage, besides the up-regulated DEGs annotated into the general function prediction only (R) and function unknown (S) classes, MULT induced up-regulated unigenes mainly grouped into the function classes: translation, ribosomal structure, and biogenesis (J) (11.5%, 7/61), secondary metabolites biosynthesis (Q) (9.8%, 6/61), and transcription (K) (6.5%, 4/61); MONO showed up-regulation unigenes mainly grouped into the transcription (K) class (3/40) (Table 1). Furthermore, the two strains made use of different transcription pathways with interactions of the respective hosts during the biotrophic stage. MULT up-regulated the unigenes encoding nascent polypeptide-associated complex subunit beta, MYB DNA-binding domain containing protein, and chromodomain helicase, while MONO induced upregulation unigenes encoding transcription factor TFIIB and transcriptional activator xlnR (Supplementary Table S8). In secondary metabolite biosynthesis, transport, and catabolism, MULT expressed relatively abundant six up-regulated unigenes including cytochrome P450, laccase precursor, L-aminoadipate-semialdehyde dehydrogenase, and ferric-chelate reductase genes to contend with stresses such as active oxygen species from hosts, while MONO expressed one up-regulated unigene encoding a ferric reductase-like transmembrane component (Supplementary Table S8). Other functional DEGs comparisons were difficult to do because of the lack of annotated MONO DEGs or poorly characterized DEGs for both strains in this phase (Supplementary Table S8).

In the last necrotrophic phase, the number of up-regulated DEGs (1,056) induced by MONO exceeded that (724) induced by MULT, changing the situation of having more MULT up-regulated DEGs than those of MONO in the invasion and biotrophic stages. It was reversed into such that there were more MONO up-regulated DGEs than those of MULT in many KOG functional classes such as in the metabolism of amino acid (E), carbohydrate (G), and lipid (I), secondary metabolites (Q), and signal transduction mechanisms (T), as well as general function prediction only (R) and function unknown (S) in this stage (Table 1). For example, the two classes of eggKOG functions in carbohydrate transport and metabolism (G) and amino acid transport and metabolism (E), containing abundant up-regulated unigenes of MONO with 46 and 34 unigenes, respectively, compared to those of MULT with 31 and 19 unigenes (Table 1), respectively. However, in the initial invasion stage, MONO induced only one and two unigenes in (G) and (E) functional classes, respectively, while MULT expressed 20 and 12 DEGs, respectively (Table 1). In this necrotrophic stage, the degrading enzymatic unigenes were also notably increased in each strain in contrast to that in the initial invasion and biotrophic phases. MULT was recorded with 27, 6, and 42 unigenes encoding degrading enzymes on carbohydrate, protein, and lipid in the three respective infection stages of initial invasion, biotrophic, and necrotrophic stages, respectively; MONO had 2, 1, and 61 unigenes in the three respective infection stages (Supplementary Table S7). Especially MONO induced 95% such unigenes in the necrotrophic infection stage (Supplementary Table S7). Moreover, in this necrotrophic stage, both strains induced abundant unigenes encoding carbohydrate enzymes including cellulase, galactosidase, glucosidase, glycoside hydrolase, pectate lyase, etc. for their rapid development. The unigenes encoding subtilisin-like protease, metalloprotease, and peptidase were dominantly expressed in protein degrading enzymes of both strains (Supplementary Table S7). The carboxylesterase and acetylxylan esterase were encoded by two lipid degrading-enzyme unigenes from the two strains in this stage (Supplementary Table S7). Moreover, MULT also induced dominant unigenes annotated in translation, ribosomal structure, and biogenesis (J) (3.2%, 22/690) and replication, recombination, and repair (L) (2.8%, 19/690); MONO did the same in secondary metabolites biosynthesis (Q) 3.8% (39/1022) (Table 1).

For common DEGs expressed through the entire infection period (Table 1), except 48 unigenes shared by the two formae speciales, dominant DEGs expressed specific to MULT were mainly involved in functions for the general function prediction only (R) (18.3%, 42/229), function unknown (S) (6.6%, 15/229), and carbohydrate transport and metabolism (4.4%, 10/229) (Table 1). MULT maintained 14 carbohydrate degrading-enzyme unigenes with up-regulated expression during its entire infection course. These unigenes encoded enzymes related to cell wall hydrolase, cutinase, pectate lyase, glucanase, polysaccharide lyase, acetylxylan esterase, mannosidase, phosphatase, etc. (Supplementary Table S9). In addition, five putative Ecp7(P20) and two metalloprotease unigenes were found to be expressed in MULT across the three infection times (Supplementary Table S9). MONO induced a total of 22 specific up-regulated expressed unigenes, only 6 of which were annotated in eggNOG and NR databases (Supplementary Table S9). Five of the six unigenes were assigned as follows: one to cell wall/membrane/envelope biogenesis (M), two to general function prediction only (R), one to transcription (K), and one to function unknown (S) (Supplementary Table S9); not any annotated unigene grouped in carbohydrate transport and metabolism function was found in common expressed DEGs of MONO (Table 1).

### *M. brunnea* Secretory Proteins and Effectors Involved in Pathogen–Host Interaction for Host-Specific Infection

**FIGURE 5:**
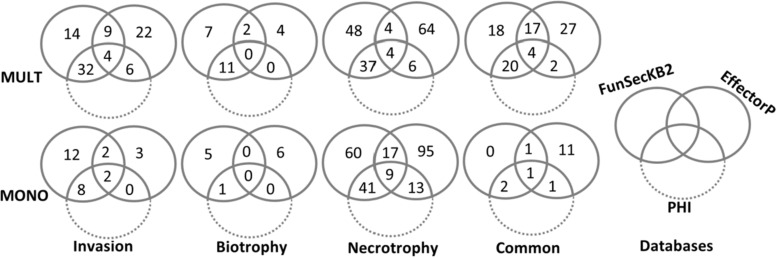
The number of up-regulated DEGs distributed with the annotations of FunSeckB2, EffectorP, and PHI databases by Venn graph.

The translated peptide sequences of the up-regulated DEG unigenes from both formae speciales against FunseckB2 database retrieved 391 fungal secretory proteins, including 230 proteins from MULT and 161 from MONO (Figure 5 and Supplementary Tables S10, S11). The same set of the up-regulated DEGs against effectorP database recovered 337 candidate effectors, including 176 and 161 from MULT and MONO, respectively (Figure 5 and Supplementary Tables S10, S11). The secretory proteins and candidate effectors were used to determine the putative effectors through overlapping between them. A total of 76 putative effector DEG unigenes were found for their hits in both the secretory proteins and the candidate effector databases with 44 unigenes from MULT and 32 unigenes from MONO (Figure 5). Among the 44 putative effectors, MULT induced 21 unigenes shared through its infective course and 13, 2, and 8 effector unigenes specific to initial invasion, biotrophic, and necrotrophic stages, respectively. MONO induced only 2 unigenes commonly expressed through its whole infective course and 4, 0, and 26 unigenes in each corresponding infection stage stated above, respectively.

The functional phenotype of the Pathogen Host Interaction database was applied for annotating the roles of DEGs encoding the putative effectors, secretory proteins, and candidate effectors (Figure 5 and Supplementary Tables S10, S11).

Based on putative effector unigenes commonly expressed through the three infection stages, MULT induced unigenes 07188 and 24657, which functioned as PHI effectors and reduced virulence, respectively (Table 2). The former unigene was a homolog of *Ecp6*, a true effector with LysM domain, which inhibited host chitinases to degrade the chitins of the pathogens *Passalora fulva* and *Fusarium oxysporum* (Supplementary Table S11). The latter was homologous to um01947 gene that functioned as putative ascorbate peroxidase to contend with toxins from host active oxygen stress by ustilago fungi (Table 2 and Supplementary Table S11). Both the MULT and the MONO strains expressed a yeast catalase gene of *CAT1*, a homologous unigene for their infections, which had an impact on virulence by PHI annotation (Table 2). In those commonly expressed putative effector unigenes without PHI annotation, MULT induced three unigenes homologous to putative Ecp7(p20), one unigene encoding phosphorylcholine phosphatase and one unigene encoding rhamnogalacturonan acetylesterase precursor with NCBI NR annotation (Supplementary Table S11). As to infection time-specific putative effectors (Table 2 and Supplementary Table S10), in the initial invasion phase, MULT forma specialis induced three up-regulated unigenes homologous to a pectate lyase, a reductase, and one hypothetical protein gene, respectively, which were annotated as virulent determinants with PHI (Table 2). MONO got one up-regulated unigene homologous to a putative Ras-related C3 botulinum toxin substrate 1, which can impact on the pathogenicity of *Magnaporthe oryzae* (Table 2 and Supplementary Table S10). In the biotrophic stage, although it had no one putative effector unigene annotated with the PHI function, MULT expressed two putative effector unigenes including a laccase and a xylosidase with NCBI NR annotation (Table 2 and Supplementary Table S10). Meanwhile, MONO had no unigenes encoding putative effectors found in the biotrophic stage. In the necrotrophic stage, MULT induced four putative effector unigenes, two of which were annotated with virulence function, including one effector gene *Ecp6* and one related to a Ras gene (Table 2). MONO had nine putative effector unigenes involved in necrotrophic infection, four of which were relevant to pathogenicity according to PHI annotation (Table 2), including one homolog to a chitinase gene of *Chi2* gene from *Metarhizium anisopliae*, one homolog to *ABA4* gene from *M. oryzae*, and two homologous genes related to pectin lyase genes (Supplementary Table S10).

**TABLE 2:** The unigenes and functions assigned as putative effectors with PHI and NR annotations.

Infection stage	Formae speciales	BMK_Unigene_ID	PHI gene	PHI function	NR annotation
Common	MU	07188	*Ecp6*	Effector	Putative Ecp7(P20)
		28518	*CAT1*	Reduced virulence	Catalase, partial
		24657	*um01947*		Putative ascorbate peroxidase
		12402	*CBP1*	Unaffected_pathogenicity	AMP-1 precursor
	Mo	28518	*CAT1*	Reduced virulence	Catalase, partial
Initial invasion	MU	14996	*PL1332*	Reduced virulence	Pectate lyase
		05917	*OXI1*		3-Ketoacyl-acyl carrier protein reductase
		07945	*GAS1*		Hypothetical protein MBM_07761
		21965	*BbbqrA*	Unaffected pathogenicity	NADH-quinone oxidoreductase
	Mo	26320	*MGG_02731__Rac1*	Loss of pathogenicity	Putative Ras-related C3 botulinum toxin substrate 1
		37034	*BCAL2200*	Unaffected pathogenicity	Phosphotyrosine protein phosphatase
Biotrophy	Mu	18091	N/A	N/A	Laccase
		12950	N/A	N/A	Beta-xylosidase, secreted
	Mo	N/A	N/A	N/A	N/A
Necrotrophy	Mu	02695	*Ecp6*	Effector	Putative Ecp7(P20)
		01791	*Fgrho4*	Reduced virulence	Ras family protein
		13777	*BgaC*	Unaffected pathogenicity	Putative beta-galactosidase
		15360	*FAEB1*	Unaffected pathogenicity	Carbohydrate esterase family 1 protein
	Mo	38072	*Chi2*	Increased virulence	–
		30822	*PL1332*	Reduced virulence	Pectate lyase
		38638	*ABA4*		Aspartyl/glutamyl-tRNA (Gln) amidotransferase, subunit B/E, catalytic
		44068	*pnl1*		Pectin lyase A precursor
		21965	*BbbqrA*	Unaffected pathogenicity, reduced virulence, increased virulence (hypervirulence)	NADH-quinone oxidoreductase
		31068	*RGSC1*	Unaffected pathogenicity	Sorting nexin-like protein
		41149	*Sch1*		Short-chain dehydrogenase
		41364	*BbCypC*		Putative peptidyl-prolyl *cis-trans* isomerase-like 3
		45896	*CPXB*		Catalase/peroxidase HPI

*N/A = no function or annotation.*

In addition to these unigenes as putative effectors annotated with the PHI function, there were many DEG unigenes encoding secretory proteins or candidate effectors annotated as pathogenicity factors with the PHI function (Supplementary Tables S12, S13). Forty-three DEG unigenes encoding secretory proteins induced by MULT specific to the early infecting period of the initial invasion and the biotrophic stages were annotated with pathogenicity or virulence factors of the PHI function (Figure 5), which included two true effectors (*PemG1* and *Mg3LysM*) and many enzyme genes for hydrolysis, redox, transfer, and synthesis (Supplementary Table S12). Only six DEG unigenes induced by MONO in the same infection period were annotated to pathogenicity or virulence factors, which were the unigenes encoding two transcript factors, two redox enzymes, an RNase, and an endopeptidase (Supplementary Table S12). Twenty-five DEGs encoding candidate effectors from MULT and MONO were annotated as pathogenicity or virulence impactors with the PHI function in three infection times (Figure 5 and Supplementary Table S13). However, only three DEGs encoding pectate lyases induced by MULT in the initial invasion were significantly recognized to impact on host specificity (Supplementary Table S13). In commonly induced DEG unigenes through the three infection phases, the unigenes encoding for two true effectors (Mg3LysM and Ecp6 with PHI gene name), two metalloproteases, four carbohydrate hydrolases for cell wall-associated carbohydrates like pectin, polysaccharide, and chitins (CcpelA, CHT42, PELB, and pnl1), three oxidoreductase (two AOX1 and one hmgA), and one serine protease (SspA-1) were found to be expressed by MULT (Supplementary Table S14). One Mg3LysM effector and one oxidoreductases (AOX1) homologous unigenes were found among MONO’s common DEG unigenes (Supplementary Table S14).

## Discussion

### The Gene Expression Profiles Showing Formae Speciales Specificity-Reflected Distinctive Host Specificity

To date, *M. brunnea* f. sp. *multigermtubi* M6 of poplar pathogens and a strain *M. coronariae* NL1 of apple pathogens (PRJNA376855, NCBI) completed their genome sequencing for fungi from *Marssonina* genus. However, the perfect state of the former was recently identified as *Drepanopeziza brunnea* (Rossman et al., 2018). The latter was identified as *Diplocarpon mali* at the perfect stage (Harada et al., 1974). Therefore, only the genome of *M. brunnea* strain M6 could be used as a reference genome for *M. brunnea* fungal transcriptome annotation, but this genome gave a significantly different reads mapping rate on the transcriptome of the MULT and the MONO formae speciales. Its annotation was able to map more than 70% unigenes for MULT, which is the same multigermtubi forma specialis as *M. brunnea* f. sp. *multigermtubi* M6, in contrast to the less than 13% unigenes mapped for MONO, which is from monogermtubi forma specialis (Supplementary Table S3). The phenomenon could reflect the fact that the monogermtubi forma specialis possessed a significant difference in functional genome from multigermtubi forma specialis during infection of their respective hosts. This discrepancy possibly originates from their various genetics associated with different host-driven forces during their evolution (Spiers, 1984; Zhu et al., 2012; Frantzeskakis et al., 2019; Morris and Moury, 2019), if it is not relevant to problematic mapping strategy. Actually, the evidences from phylogenetical analyses have robustly distinguished them as different lineages (Han et al., 1998b, 2000; Sun et al., 2015).

Furthermore, a *de novo* transcriptome was assembled for transcriptomic comparison between formae speciales MULT and MONO in this study. The results showed that the gene expression profiles between MULT and MONO could be characterized with a V-shape change and a mirror L-shape change for their infections (Figure 4C), although they performed very similarly in terms of the infection process (Spiers, 1989; Zhang et al., 2018a). MULT induced more than 40% DEGs in the initial invasion stage and MONO expressed less than 9% DEGs in the invasion stage (Figure 4). The abundant and diverse expressed genes in the early invasion stage assure MULT to accommodate or infect successfully more diverse poplar hosts than MONO (Frantzeskakis et al., 2019; Morris and Moury, 2019). In nature, *Populus* sections Aigeiros and Tacamahaca host most modern species, abundant with genetically modified varieties or highly hybridized species for woody crops with industrial, ecological, and bio-energy purposes (McKown and Guy, 2018; Zanewich et al., 2018; Zhang et al., 2019). These diverse host selective forces from diverse species hosts possibly drove multigermtubi forma specialis to evolve more diverse pathogen-associated molecular patterns (PAMPs) and effectors for dismissing the evolving diverse host pattern recognition receptors (PRRs) and R genes defenses (Nurnberger and Kemmerling, 2009; Frantzeskakis et al., 2019). The poplar hosts in section Leuce of monogermtubi fungi were also earlier lineage with low abundant species and few intrasectional crossed hybridized varieties in a slow evolutionary pace compared to other subgenus poplars in *Populus* genus (Christe et al., 2016; Zhang et al., 2019). Monogermtubi fungi also were in earlier evolution than the multigermtubi fungi (Han et al., 1998b, 2000; Sun et al., 2015). Therefore, monogermtubi forma specialis could maintain pathogenicity in an inflexible gene expression way for coping with the early interaction infecting its hosts compared to the multigermtubi one (Figure 4). Furthermore, MULT induced 27 degrading enzyme unigenes, while MONO induced only 2 degrading enzyme unigenes in the initial invasion and biotrophic stages (Supplementary Table S7). The degrading enzymes, especially carbohydrate-degrading enzymes such as pectate lyases, cell wall hydrolases, glucanases, etc. were crucial factors for pathogenicity in the colonization and the early invasion (Yuan et al., 2008; Gan et al., 2013; Ma et al., 2015). The abundant and diverse degrading enzymes make a pathogen infect diverse hosts (Gan et al., 2013; Morris and Moury, 2019). These enzyme expressions and activities affect the pathogenic virulence during an infection (Gan et al., 2013; Ma et al., 2015) and impact host ranges and host specificity (Shirke et al., 2016; Borah et al., 2018; Morris and Moury, 2019). At least the pectate lyase and metalloprotease could play a much crucial role in the pathogenesis of a multigermtubi fungus on the hosts (Supplementary Table S14; Chen et al., 2015). Actually, the responses of their own susceptible hosts during an infection consistently performed in gene expression profile at the genome level (Zhu et al., 2012; Chen et al., 2015; Xing et al., 2018; Zhang et al., 2018b). *P. deltoids*, a host from section Aigeiros, induced the most abundant DEG at initial invasion with a multigermtubi fungus challenge (Zhang et al., 2018b). *P. alba*, a host from section Leuce, responded with the most abundant DEGs at the necrotrophic stage to a monogermtubi fungus infection (Zhang et al., 2018b). The unique pattern of the specific DEG’s expression of *Marssonina* formae speciales could have stemmed on the co-evolutionary forces from its own poplar hosts (Christe et al., 2016; Frantzeskakis et al., 2019; Taylor and Larson, 2019; Zhang et al., 2019). Interestingly, nearly half a hundred of DEGs expressed in the initial invasion and biotrophic stages to MULT were discovered to have their homologous or same unigenes expressed in the necrotrophic stage to MONO with the same NCBI-NR function annotation (Supplementary Table S15). For instance, MULT expressed degrading enzyme DEGs (nine kinds of unigenes in the initial invasion stage and one kind in the biotrophic stage) related to carbohydrate, lipid, and protein metabolism for its early colonization and infection, which were not found to be expressed in MONO at the initial invasion and biotrophic stages (Supplementary Table S15). However, all of their homologs or the same unigenes were induced in the necrotrophic stage by MONO (Supplementary Table S15). This evidence showed that the infection transcriptomes between the two formae speciales appeared at a lagged time in the phytopathogenic molecular process. Therefore, the unique gene expression profiles of MULT and MONO reflected their respective host specificity in the interaction level.

### The Roles of Effectors in Forma Specialis Specificity-Reflected Distinctive Host Specificity

The roles of effectors in host-specific interactions have been evident with more and more demonstrations on genomic comparisons and effector gene functions (Shirke et al., 2016; Inoue et al., 2017; Borah et al., 2018; Morris and Moury, 2019). The absence of putative-secreted effector protein genes made *Melanopsichium pennsylvanicum* parasitic dicot plants of the *Persicaria* genus (Sharma et al., 2014). The function loss of disrupted PWT3 made *M. oryzae* infect a new variety of wheat (Inoue et al., 2017). In this study, 10 true effector genes annotated with the PHI function were found in common or time-specific up-regulated DEG expression in MULT and MONO infection processes, including six LysM domain *Ecp6* and *Mg3LysM*, two *PemG1*, one *XEG1*, and one *ACE1* (Table 3 and Supplementary Tables S10, S11). *Mg3LysM* effectors are involved in binding peptidoglycan or chitin to prevent host chitin-triggered immunity (Akcapinar et al., 2015). However, only one unigene (23925) encoding *Mg3LysM* was found as commonly expressed in MONO (Table 3). Two other Mg3LysM unigenes (23925 and 03333) were specifically induced by MULT in the invasion stage and common through the infection course, respectively (Table 3). The three Ecp6 unigenes were induced specifically by MULT with two unigenes (07188 and 07333) commonly expressed through the infection course and one unigene (02695) induced in the necrotrophic stage (Table 3). Furthermore, there were five unigenes annotated as putative Ecp7(p20) of LysM domain genes by the NCBI NR database which were induced specifically in common through the whole infection course or in the necrotrophic stage by MULT. Their pathogenicity on host specificity needed to be further verified (Table 3), but putative LysM domain genes were recorded to be important pathogenic factors of *M. brunnea* f. sp. *multigermtubi* fungi in their host poplars (Yuan et al., 2008; Cheng et al., 2010; Zhu et al., 2012; Jiang et al., 2014; Chen et al., 2015). This study showed that *Mg3LysM*, not *Ecp6* effector genes, were important to the pathogenicity of monogermtubi forma specialis. Furthermore, individual LysM domain effector genes are able to perform very specific carbohydrate binding in many cases, resulting in a unique recognition interaction in the pathosystem (Akcapinar et al., 2015; Inoue et al., 2017; Borah et al., 2018; Frantzeskakis et al., 2019; Morris and Moury, 2019). Therefore, the effector protein ortholog to Ecp6 could be a determinant factor in host specificity to the two formae speciales diversification in *M. brunnea*.

**TABLE 3:** The unigenes functioned as true effectors with PHI and NR annotation.

Infection stage	Forma specialis	BMK unigene	NR annotation	PHI annotation			
				Gene	Function	Species	*E*-value
Invasion	MULT	23925	Putative cell wall-associated hydrolase	*Mg3LysM*	Effector	*Zymoseptoria tritici*	5.00E-20
Biotrophy	MULT	08152	Hypothetical protein MBM_08839	*PemG1*	Effector	*Magnaporthe oryzae*	1.00E-71
Necrotrophy	MONO	45143	Endoglucanase	*XEG1*	Effector	*Phytophthora sojae*	2.00E-21
	MONO	45853	Hypothetical protein CFIO01_09885	*ACE1*	Effector	*Magnaporthe oryzae*	e-139
	MULT	09776	Hypothetical protein V500_00134	*PemG1*	Effector	*Magnaporthe oryzae*	0.000008
	MULT	02695	Putative Ecp7(P20)	*Ecp6*	Effector	*Passalora fulva*, *Fusarium oxysporum*	8E-09
	MULT	03009	Putative Ecp7(P20)	N/A	N/A	N/A	N/A
	MULT	03228	Putative Ecp7(P20)	N/A	N/A	N/A	N/A
Common	MULT	07188	Putative Ecp7(P20)	*Ecp6*	Effector	*Passalora fulva*, *Fusarium oxysporum*	0.000006
	MULT	04762	Putative Ecp7(P20)	N/A	N/A	N/A	N/A
	MULT	03019	Putative Ecp7(P20)	N/A	N/A	N/A	N/A
	MULT	13082	Putative Ecp7(P20)	N/A	N/A	N/A	N/A
	MULT	07333	Hypothetical protein MBM_08215	*Ecp6*	Effector_	*Passalora fulva, Fusarium oxysporum*	0.00001
	MULT	03333	Putative cell wall-associated hydrolase	*Mg3LysM*	Effector	*Zymoseptoria tritici*	3.00E-19
	MONO	23925	Putative cell wall-associated hydrolase	*Mg3LysM*	Effector	*Zymoseptoria tritici*	5.00E-20

*N/A = no annotation.*

The other four true effector genes of two *PemG1*, a *XEG1*, and an *ACE1* could also take roles in host specificity to *M. brunnea*. The two expressed *PemG1* orthologs (unigenes 08152 and 09776) occurred at the biotrophic stage and through the infection course, respectively, specifically in MULT but not in MONO (Table 3). The PemG1 effector protein is a common elicitor of pathogens in recognition interaction with the plant host (Qiu et al., 2009). Orthologs to *XEG1* and *ACE1* were induced specific to MONO’s necrotrophic infection (Table 3). XEG1 is a glycoside hydrolase with xyloglucanase activity and is an apoplastic elicitor of cell death (Ma et al., 2015). *ACE1* encodes a putative hybrid between a polyketide synthase and a non-ribosomal peptide synthetase and is expressed exclusively during the fungal penetration of host leaves (Böhnert et al., 2004). These *XEG1* and/or *ACE1* orthologs could take roles in nutrients for the pathogen to grow. They were not induced with the infection of MULT forma specialis. Therefore, these unique secretory proteins and effectors mentioned above could be among the important determinants of host specificity for the two formae speciales. No previous evidence is available for analyses on the pathogenicity of forma specialis monogermtubi in the molecular and the genome level. This study provided the first glance on molecular interaction evidences with host for the *M. brunnea* f. sp. monogermtubi fungi. Furthermore, many DEG unigenes encoding putative effectors or secretory proteins for both formae speciales, especially to MONO, were not annotated with nucleotide or protein databases at present (Figure 5 and Supplementary Tables S11–14). These unigenes could provide splendid putative functional genes for further exploration of the characterizing nature of host specificity. Finally, this study made a hypothetical protein MBM_08215 from the genome of *M. brunnea* f. sp. *multigermtubi* M6 as a new Ecp6 effector (Table 3). Also, this study attributed three M6 strain’s sequences of hypothetical protein MBM_08839, V50000134, and CFIO01_09885 into PemG1 and ACE1 effectors. Besides the true effector genes with PHI annotation, secretory protein and candidate effector (excluding putative effector) unigenes expressed in the early infection stages of invasion and biotrophy also make contribution to host-specific activities between the two formae speciales (Supplementary Tables S10, S11).

## Conclusion

The variation in genome-level transcription among individuals can contribute to the explanation on the pathogenic virulent aspects of the host specificity diversity from pathogenic virulent aspects (Clark and Townsend, 2007; Crawford and Oleksiak, 2007; Cheng et al., 2012). This report presented not only a better understanding on the contribution of fungal genome to host specificity *via M. brunnea* virulence differential formae speciales but also novel knowledge on gene expression in the genome level for the forma specialis *M. brunnea* f. sp. *monogermtubi*. Interestingly, the two formae speciales employed significantly different sets of virulence-related genes encoding effectors and other secretory proteins to infect their own susceptible hosts and have a significantly differential molecular procedure to infect their own susceptible hosts at the infection time course. The evidences pointed out that genes related to virulence could drive or have major roles to evolve *Marssonina* populations into the two diversification groups. In view of *M. brunnea* fungi as a typical hemibiotrophic pathogen only infecting wood crop poplars, our knowledge could facilitate the development of a model for exploring host specificity in woody plants with hemibiotrophic fungal pathogens with detailed poplar genome biology (Jiang et al., 2019).

## Data Availability Statement

The datasets generated for this study can be found in the Bioprojects accession numbers PRJNA395840 in NCBI.

## Author Contributions

D-HY conceived the funds and conceptualized and designed the experiments, and wrote the manuscript. FR, GW, XMS, and XS performed the experiments. FR and D-HY analyzed the data. D-HY and RL contributed reagents, materials and analysis tools.

## Conflict of Interest

The authors declare that the research was conducted in the absence of any commercial or financial relationships that could be construed as a potential conflict of interest.

## Footnotes

1 http://www.phi-base.org

## Abbreviations

DEGs: differentially expressed unigene (gene)
hpi: hours post-inoculation
MONO: a strain from *Marssonina brunnea* f. sp. *Monogermtubi*
MULT: a strain from *Marssonina brunnea* f. sp. *multigermtubi*
qRT-PCR: quantitative reverse transcription-PCR.
