# Supplementary material for: Distinctive Gene Expression Profiles and Effectors Consistent With Host Specificity in Two Formae Speciales of Marssonina brunnea
Source: Front Microbiol. 2020 Mar 10;11:276. doi: 10.3389/fmicb.2020.00276 (PMC7076119; doi:10.3389/fmicb.2020.00276)
Supplement: Supplementary file 16 [file Data_Sheet_1.pdf]

Supplementary Material

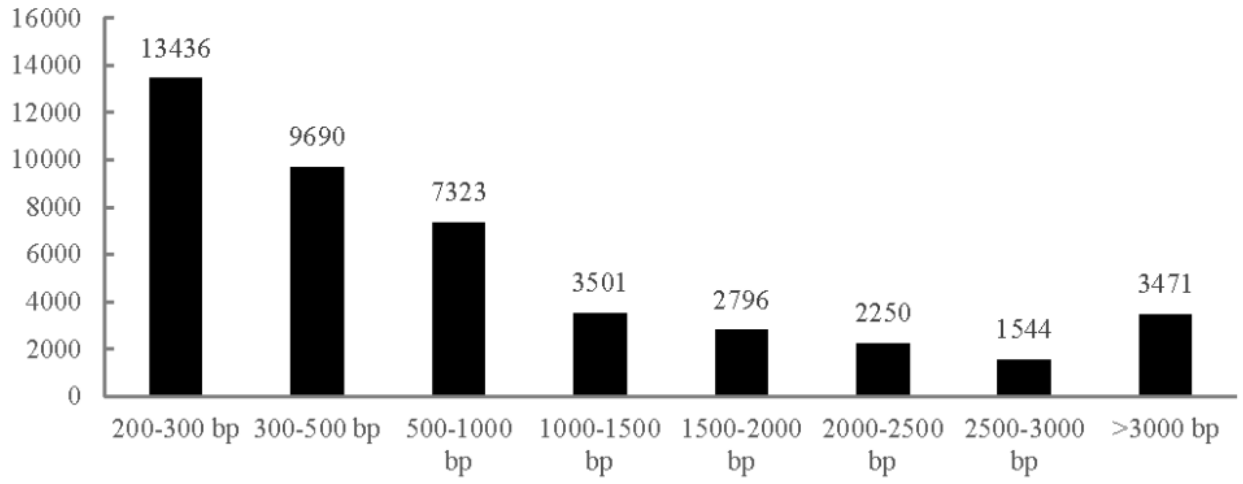

Figure S1. The number distribution with assembled sequence length for the assembled sequence quality of the *de novo* transcriptome with the two *M. brunnea* formae species. The *de novo* assembly resulted in 44,011 unigenes in length ranges from 200 bp to more than 3,000 bp with a mean length of ca. 1,042 bp, N50 length of 2,151 bp.

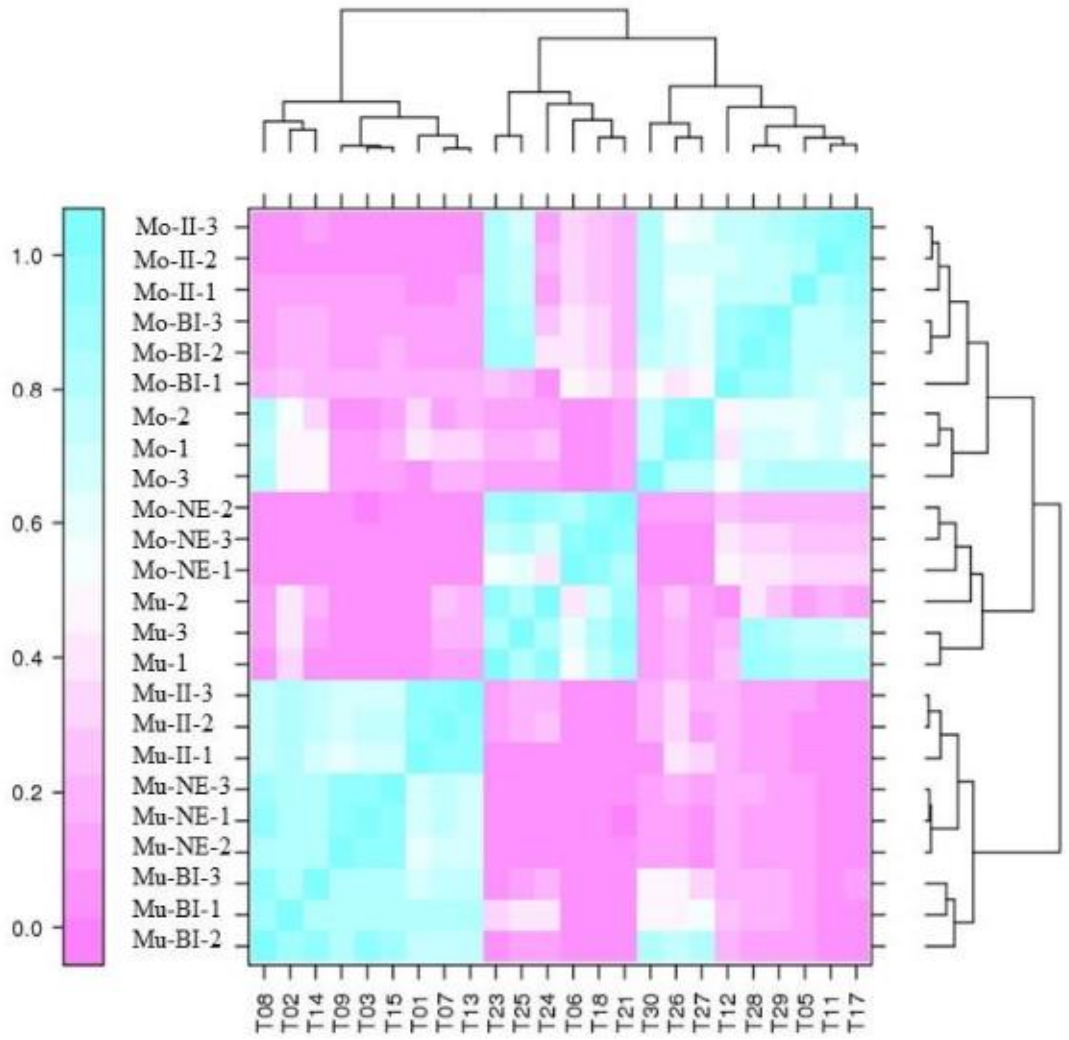

Figure S2: the heatmap of transcripts for all samples with phylogeny to show samples cluster. MULT triplicate samples in initial invasion stage: T01: Mu-II-1, T07: Mu-II-2, T13: Mu-II-3; MULT triplicate samples in biotrophic stage: T02: Mu-BI-1, T08: Mu-BI-2, T14: Mu-BI-3; MULT triplicate samples in necrotrophic stage: T03: Mu-NE-1, T09: Mu-NE-2, T15: Mu-NE-3; MULT triplicate samples of conidial control: T23: Mu-1, T24: Mu-2, T25: Mu-3; MONO triplicate samples in initial invasion stage: T05: Mo-II-1, T11: Mo-II-2, T17: Mo-II-3; MONO triplicate samples in biotrophic stage: T12: Mo-BI-2, T28: Mo-BI-2, T29: Mo-BI-3; MONO triplicate samples in necrotrophic stage: T06: Mo-NE-1, T18: Mo-NE-2, T21: Mo-NE-3; MONO triplicate samples of conidial control: T26: Mo-1, T27: Mo-2, T30: Mo-3.

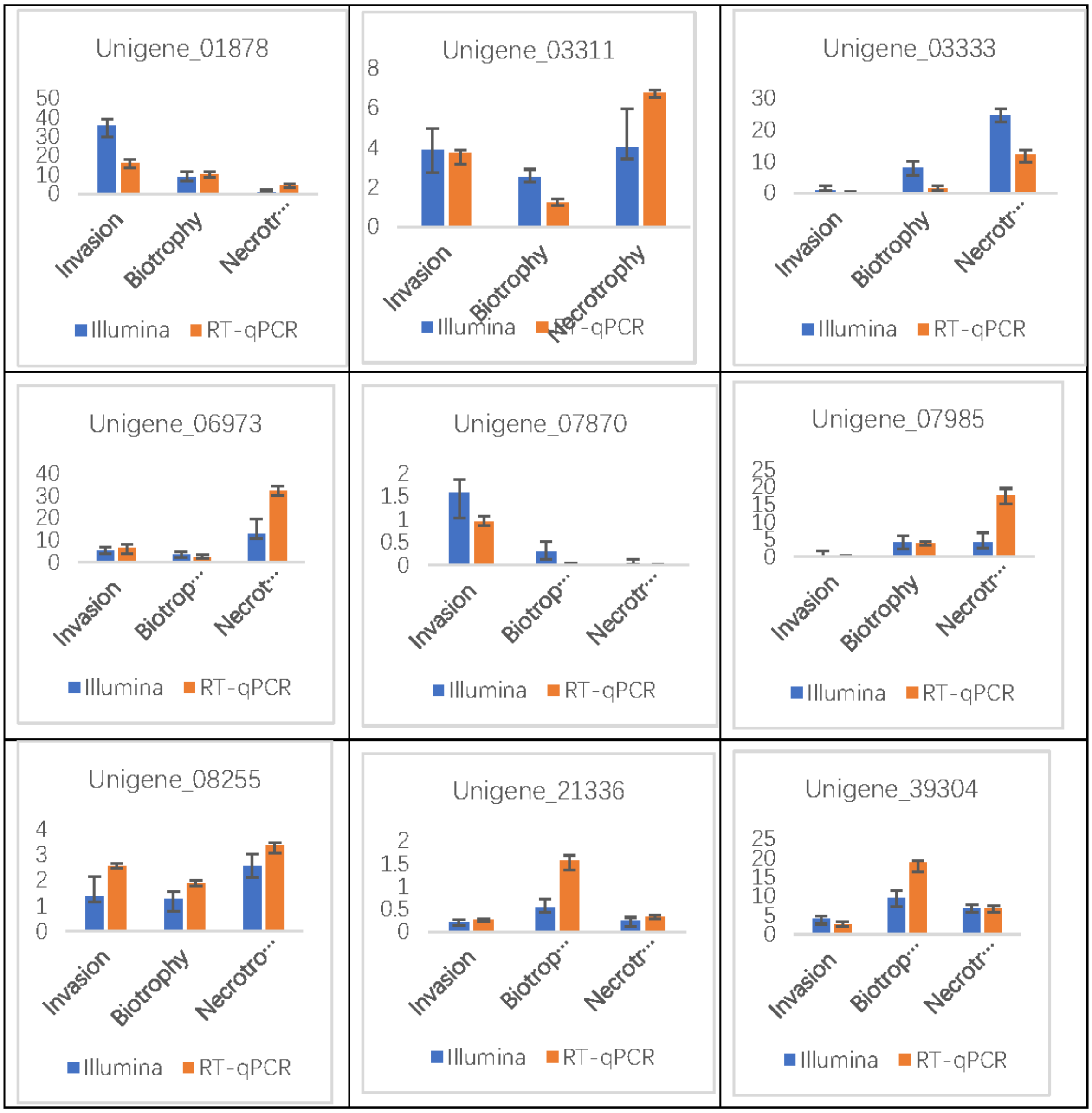

Figure S3. Validation on transcriptome by qRT-PCR with elected expressed unigenes. The molecular functions of the unigenes_01878, 03311,03333, 06973, 07870, 07985, 08255, 21336 and 39304 with NCBI-NR annotation: conserved protein, endoplasmic Reticulum Oxidoreductin 1, putative cell wall-associated hydrolase, hypothetical protein MBM_02340, hypothetical protein MBM_05641, 40S ribosomal protein S29, cell wall glucanase, cobalamin-independent methionine synthase and no annotation.
